# Analysis of risk factors for post-bacillus Calmette–Guerin-induced prostatitis in patients with non-muscle invasive bladder cancer

**DOI:** 10.1038/s41598-020-66952-4

**Published:** 2020-06-17

**Authors:** Tae Jin Kim, Young Dong Yu, Sung Il Hwang, Hak Jong Lee, Sung Kyu Hong, Sang Eun Lee, Jong Jin Oh

**Affiliations:** 1 Department of Urology, CHA University College of Medicine, CHA Bundang Hospital, Seoul, Korea; 2 Department of Radiology, Seoul National University Bundang Hospital, Seoul, Korea; 3 Department of Urology, Seoul National University College of Medicine, Seoul National University Bundang Hospital, Seoul, Korea

**Keywords:** Cancer, Oncology, Risk factors, Urology

## Abstract

The objective of this study was to evaluate risk factors for bacillus Calmette–Guerin-induced prostatitis in patients with non-muscle invasive bladder cancer following bacillus Calmette–Guerin therapy. Clinical findings from patients with non-muscle invasive bladder cancer who underwent multi-parametric magnetic resonance imaging before transurethral resection of bladder tumor and post-bacillus Calmette–Guerin therapy from March 2004 to August 2018 were evaluated. The population was grouped into patients with or without newly developed lesions on multi-parametric magnetic resonance imaging performed 3 months after bacillus Calmette–Guerin instillation. Patients with prostate-specific antigen levels ≥ 4 ng/mL or prostate cancer were excluded. Univariable and multivariable analyses were performed to determine the predictors of prostate lesions in patients with prior bacillus Calmette–Guerin exposure. Post bacillus Calmette–Guerin-induced prostatitis was found in 50 of the 194 patients (25.8%). No significant differences were observed between the groups except for prostate volumes (33.8 mL vs. 30.8 mL, P = 0.012) and body mass index (25.2 kg/m^2^ vs. 24.1 kg/m^2^, P = 0.044). After bacillus Calmette–Guerin exposure, no significant differences in prostate-specific antigen levels, international prostate symptom scores, or post-voiding residual volume were noted. Multivariable regression analysis showed that body mass index (odds ratio, OR = 1.115, P = 0.038) and prostate volume (OR = 3.080, P = 0.012) were significant predictors of post-bacillus Calmette–Guerin prostate lesions. Body mass index and prostate volume may be clinical predictors of prostate lesions after bacillus Calmette–Guerin exposure. Awareness of potential risk factors for this entity should contribute to the clinical decision-making process for patients following bacillus Calmette–Guerin therapy.

## Introduction

Intravesical treatment of bacillus Calmette–Guerin (BCG) has become a standard modality to reduce the risk of recurrence and progression of superficial non-muscle invasive bladder cancer (NMIBC)^1^. Although histological evidence of granulomatous prostatitis (GP) is a common manifestation in BCG administered patients^2^, the majority of these patients are asymptomatic without serious clinical complications^3^. Nevertheless, a significant increase in prostate-specific antigen (PSA) levels in up to 40% of cases may be observed^4^.

Even though BCG-related local complications after intravesical BCG therapy are well studied, the possibility of systemic infections and other severe adverse events must be considered^3^.

In patients with NMIBC following BCG therapy, multi-parametric magnetic resonance imaging (mpMRI) is typically done to identify extravesical involvement. Acute BCG-induced GP may show findings similar to those of prostate cancer on mpMRI. Therefore, in patients with abnormal mpMRI findings after BCG instillation, the clinical decision to initiate treatment with tuberculosis mediation is challenging. Evidence and study results on the occurrence, pathogenesis, and management of abnormal prostate mpMRI findings resulting from intravesical BCG administration have been sporadically available;^2–4^ however, no studies on the risk factors for this entity have yet been undertaken.

Therefore, in this study, we investigated the clinical characteristics and analyzed potential prognostic risk factors for abnormal mpMRI prostatic lesions in NIMBC patients exposed to intravesical BCG therapy.

## Methods

### Patients

**Figure 1:**
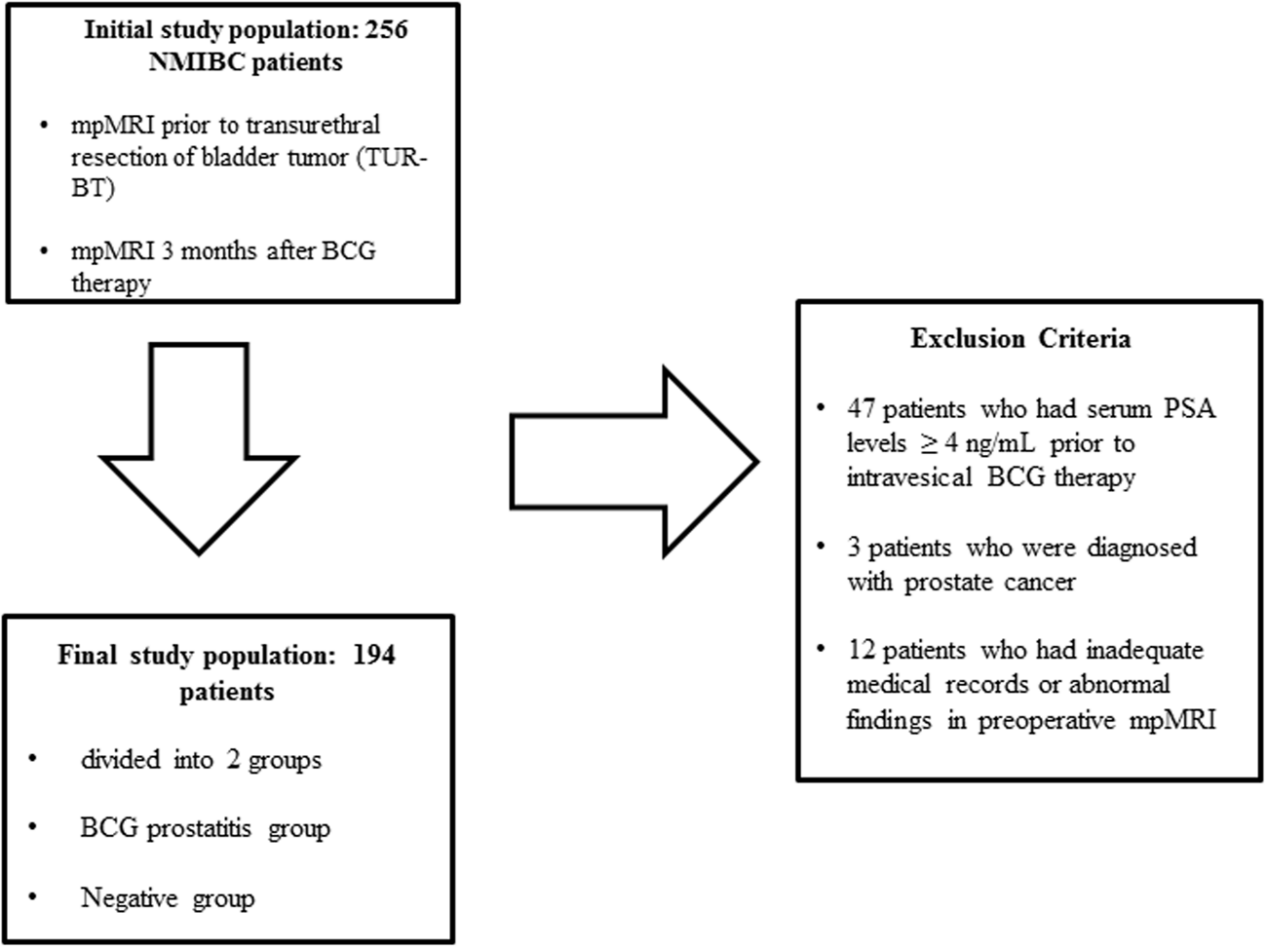
Patient selection and exclusion criteria.

The medical records of 256 patients who underwent mpMRI prior to transurethral resection of bladder tumor (TUR-BT) and after BCG therapy for NMIBC between March 2004 and August 2018 were retrospectively reviewed. Magnetic resonance imaging was routinely done to all candidates for a preoperative evaluation and follow up study purposes 3 months after intravesical BCG instillation. Despite having no abnormal findings in prior MRI imaging, 47 patients who had serum PSA levels ≥ 4 ng/mL prior to intravesical BCG therapy were excluded due to the possibility of prostate cancer^5^. Another 3 patients who were diagnosed with prostate cancer and 12 patients who had inadequate medical records or abnormal radiologic findings in the preoperative mpMRI scans were also excluded. Among the remaining 194 patients, we divided the study population into 2 groups, the BCG prostatitis group and the negative group, to compare the incidence of prostatic lesion development after BCG exposure (Fig. 1).

**Figure 2:**
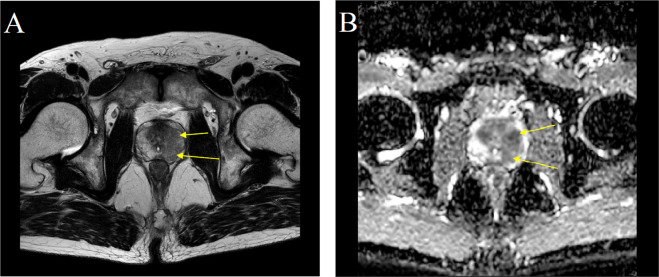
mpMRI of a male patient with stage T1 high-grade bladder cancer 3 months after intravesical BCG. (**A**) T2-weighted MRI demonstrating focal circumscribed nodules of decreased signal involving the transitional zone and left peripheral zone (arrows). (**B**) Apparent diffusion coefficient map demonstrating a decreased signal corresponding to the T2-weighted images (arrows).

In this study, we defined BCG prostatitis as a homogenous, moderately hypointense focus/mass confined to the prostate with a corresponding decreased signal in apparent diffusion coefficient (ADC) maps on mpMRI scans obtained 3 months after BCG instillation (Fig. 2). Clinical and pathological parameters including age, body mass index (BMI), clinical history of hypertension and diabetes mellitus, pathological stage and grade of bladder cancer, presence of concomitant carcinoma *in situ* (CIS), serum PSA levels, post-voiding residual volume (PVR), international prostate symptom scores (IPSS), and prostate volume were reviewed. In addition, radiological and clinical findings after intravesical BCG therapy were reviewed. We evaluated the clinical courses and risk factors for BCG therapy patients with abnormal radiologic findings of the prostate gland.

### Intravesical BCG administration and Prostate biopsy

Patients who underwent TURBT and had histopathological confirmation of intermediate and high risk NMIBC were administered intravesical BCG at 2 weeks after surgery. A follow up urinalysis and culture was done prior to BCG therapy to exclude patients with conventional urinary tract infections (UTI) and there were no patient symptoms of urinary tract infection or hematuria before BCG instillation. Weekly administration of BCG therapy was scheduled for 6 weeks for stage Ta-T1 tumors and 8 weeks for CIS at a dose of 50 mg of Onco Tice strain in 50 mL saline instilled into the bladder using an 8 Fr urethral catheter, with retention for 1–2 hours. Patients with abnormal mpMRI findings done after intravesical BCG instillation or those presenting BCG side effects and clinical symptoms underwent transrectal ultrasound (TRUS) guided biopsy for further evaluation and management.

### mp MRI imaging protocol

Prostate mpMRI was performed at 3-T magnetic field strength with a pelvic phased-array coil. T1-, T2-, and diffusion-weighted imaging sequences were attained. The median interval from mpMRI to TUR-BT was 3.1 weeks (range, 0.0–15.3). The median interval time from TUR-BT to BCG administration was 1.5 months (range, 0.4–5.3), and the median interval from BCG therapy to follow up mpMRI was 3.1 months (range, 0.6–11.2). Every MRI scan was interpreted by an experienced radiologist in a clinical setting who was not blinded to the clinical context. Lesions were determined to be in the peripheral zone (PZ) or transition zone (TZ) based on the T2-weighted sequences and were evaluated using the Prostate Imaging Reporting and Data System (PI-RADS) v2 guidelines according to their respective locations.

### Statistical analysis

Statistical analysis was performed using IBM SPSS Statistics Version 21 (IBM, New York, NY, USA). Factors evaluated for association with a positive biopsy included age, PSA level, clinical history, prostate volume, BMI, and PVR. Univariate and multivariate analyses with logistic regression were used to identify the significant predictors of BCG prostatitis. Hazard ratios and 95% confidence intervals (CIs) were determined. Values of P < 0.05 were considered significant. This study was conducted in accordance with the guidelines of the Declaration of Helsinki and approved by our Ethical Committee.

### Ethical approval

Informed consent was obtained from all patients and all procedures performed in the study involving human participants were in accordance with the ethical standards of the institutional and/or national research committee (Seoul National University Bundang Hospital Institutional review board; protocol B-2001-586-108) and with the 1964 Helsinki declaration and its later amendments or comparable ethical standards.

## Results

### Baseline characteristics

The mean age of the BCG prostatitis group was 66.2 ± 11.3 years, and the mean age of the negative group was 67.2 ± 10.8 years; the pre-BCG instillation PSA levels of each group were 1.87 ± 1.49 ng/mL and 1.55 ± 1.92 ng/mL, and the estimated prostate volumes were 33.8 ± 10.7 mL and 30.8 ± 11.7 mL, respectively. The mean total IPSS was 17.93 ± 3.79 in the BCG prostatitis group and 16.18 ± 5.46 in the negative group, while the mean PVR was 35.3 ± 5.2 mL and 32.5 ± 4.9 mL, respectively. The mean BMI was 25.2 ± 3.5 kg/m^2^ in the BCG prostatitis group and 24.1 ± 3.3 kg/m^2^ in the negative group (Table 1). There were 35 (70.0%) stage T1 cases in the BCG prostatitis group and 107 (74.3%) in the negative group. Thirty-six patients (72.0%) in the BCG prostatitis group and 46 patients (31.9%) in the negative group showed a G3 element. Concomitant CIS lesions were detected in 3 patients in the BCG prostatitis group and 12 patients in the negative group. Table 1 presents the details of the histopathological characteristics of the total study population.

**Table 1:** Patient baseline and histopathological characteristics prior to BCG therapy.

*Parameters*	BCG prostatitis (n = 50)	Negative (n = 144)	P-value
Age, years, mean ± SD	66.2 ± 11.3	67.2 ± 10.8	0.580
BMI, kg/m^2^, mean ± SD	25.2 ± 3.5	24.1 ± 3.3	0.044
Diabetes mellitus (%)	11 (22.0)	32 (16.5)	0.974
Hypertension (%)	23 (46.0)	71 (49.3)	0.688
History of smoking (%)	39 (38.0)	99 (68.8)	0.205
PSA, ng/mL, mean ± SD	1.87 ± 1.49	1.55 ± 1.92	0.238
IPSS, mean ± SD	17.93 ± 3.79	16.18 ± 5.46	0.312
Prostate volume, cc, mean ± SD	33.8 ± 10.7	30.8 ± 11.7	0.012
Post-voiding residual volume, mL, mean ± SD	35.3 ± 5.2	32.5 ± 4.9	0.135
Pathologic characteristics			
Tumor grade (%)			0.370
G1	3 (6.0)	10 (6.9)	
G2	11 (22.0)	88 (61.1)	
G3	36 (72.0)	46 (31.9)	
Pathological T stage (%)			0.723
Ta	12 (24.0)	29 (20.1)	
Tis	3 (6.0)	8 (5.6)	
T1	35 (70.0)	107 (74.3)	
Concomitant CIS	3 (6.0)	12 (8.3)	0.585

BCG, bacillus Calmette–Guerin; BMI, body mass index; PSA, prostate-specific antigen; IPSS, International Prostate Symptom Score; CIS, carcinoma *in situ*; OR, odds ratio; CI, confidence interval; SD, standard deviation.

### Clinical and radiological findings after BCG administration

Table 2 summarizes the radiological and clinical findings 3 months after BCG instillation therapy. No significant differences between the patient characteristics were observed 3 months after intravesical BCG treatment, including serum PSA levels, IPSS scores, and PVR. Abnormal findings on the MRI scans were detected in the prostate glands of 50 (25.8%) of the 194 patients examined after BCG therapy. PZ involvement accounted for 66% of the cases, while TZ lesions were identified in 34% of the patients with GP. Twenty-nine patients had a PI-RADS score of ≤3, while 26% of the patients had PI-RADS 4 lesions and the remaining 16% had a PI-RADS score of 5. Thirteen patients who either showed abnormal mpMRI findings after BCG instillation or presented clinical symptoms underwent transrectal ultrasound (TRUS) guided biopsy. Of the 13 patients, 4 cases (30.7%) were diagnosed with granulomatous prostatitis. Histopathology results showed no patients with prostate cancer. After stratifying these patients into subgroups (9 patients in the PI-RADS ≤ 3 group, and 4 patients in PI-RADS > 3 group), the diagnostic rate for granulomatous prostatitis was 75% (3 cases) in the PI-RADS > 3 group and 11.1% (1 case) in the PI-RADS ≤ 3 subgroups.

**Table 2:** Radiological findings and clinical findings 3 months after BCG instillation therapy.

*Parameters*	BCG-induced prostatitis (n = 50)	Negative (n = 144)	P-value
Radiological characteristics after BCG therapy			
Location of lesion			
Peripheral zone	33 (66.0)		
Transitional zone	17 (34.0)		
PIRADSv2 score			
1	5 (10.0)		
2	14 (28.0)		
3	10 (20.0)		
4	13 (26.0)		
5	8 (16.0)		
Clinical characteristics after BCG therapy			
PSA, ng/mL, mean ± SD	3.80 ± 3.44	2.48 ± 3.22	0.314
IPSS, mean ± SD	18.59 ± 6.07	17.32 ± 5.74	0.241
Post-voiding residual volume, cc, mean ± SD	49.36 ± 8.43	31.85 ± 6.70	0.324

BCG, bacillus Calmette–Guerin; PSA, prostate-specific antigen; IPSS, International Prostate Symptom Score; OR, odds ratio; CI, confidence interval; SD, standard deviation.

Side effects occurred in 65 patients (33.5%) after intravesical BCG instillation (Table 3). Three patients from the prostatitis group developed fever and 10 cases were noted in the negative group. Hematuria occurred in 21 patients (5 cases in the prostatitis group and 16 in the negative group), LUTS was found in 5 patients from the prostatitis group and 13 patients showed symptoms in the negative group. Patients presenting other side effects were 12% (2 cases of epididymorchitis, 3 cases of nausea and 1 case of malaise) and 4.9% (1 case of epididymorchitis, 4 cases of nausea, 1 case of malaise and 1 case of abdominal ileus) respectively. No significant differences in the presence or absence of any adverse effects of BCG therapy were observed between patients with and without abnormal radiological findings of the prostate gland after BCG therapy.

**Table 3:** Comparison of the occurrence of intravesical BCG administration side effects.

*Parameters*	BCG prostatitis(n = 50)	Negative(n = 144)	P-value
Asymptomatic	31 (62%)	98 (68.1%)	0.511
Fever	3(6%)	10 (7%)	0.497
Hematuria	5(10%)	16 (11.1%)	0.155
LUTS	5 (10%)	13 (9%)	0.382
Other side effects	6 (12%)	7 (4.9%)	0.781

BCG, bacillus Calmette–Guerin; LUTS, Lower urinary tract symptoms.

### Predictors of BCG induced prostatitis

The multiple logistic regression analysis for the predictors of GP is summarized in Table 4. Univariable logistic regression analysis showed that BMI (P = 0.048) and prostate volume (P = 0.008) were significant predictors of GP. Multivariable logistic regression analysis revealed that BMI (P = 0.038) and prostate volume (P = 0.012) were independent predictors of BCG-induced prostatitis.

**Table 4:** Logistic regression analysis to determine the factors predictive of BCG-induced granulomatous prostatitis.

*Parameters*	Univariable factors		Multivariable factors	
	OR (95% CI)	P-value	OR (95% CI)	P-value
Age	0.992 (0.963–1.021)	0.578	0.975 (0.911–1.043)	0.467
BMI	1.104 (1.001–1.217)	0.048	1.115 (1.009–1.231)	0.038
Hypertension	0.876 (0.460–1.669)	0.687	0.971 (0.522–2.435)	0.818
Diabetes mellitus	0.987 (0.454–2.145)	0.974	0.561 (0.108–2.908)	0.561
Smoking history	1.612 (0.757–3.433)	0.216	1.394(0.551–3.527)	0.483
PSA	1.001(0.777–1.289)	0.076	0.916 (0.782–1.073)	0.099
Prostate volume	3.267 (1.370–7.794)	0.008	3.080 (1.238–7.406)	0.012
Post-voiding residual volume	1.018 (1.000–1.036)	0.082	1.008 (0.994–1.022)	0.065
IPSS	1.003(0.992–1.012)	0.481	0.968 (0.904–1.037)	0.359

BCG, bacillus Calmette–Guerin; BMI, body mass index; PSA, prostate-specific antigen; IPSS, International Prostate Symptom Score; OR, odds ratio; CI, confidence interval.

## Discussion

Secondary prostatitis after BCG instillation into the bladder for the management of NMIBC may have local and systemic complications, which include prostatic abscess and GP. According to previous histopathological reports^2,6^, the majority of patients who underwent BCG immunotherapy developed granulomatous inflammatory lesions in the prostate. Most patients are asymptomatic, and only a low rate (0.9–1.3%) of clinical complaints such as slight induration of the prostate or elevated PSA levels has been reported^3,4^. In these patients, the radiologic findings may exhibit similarities to those of prostate cancer^7^, for which a prostate biopsy may be required to determine the diagnosis^3^.

While the pathogenesis of BCG-induced prostatitis is uncertain, it is often a prostatic inflammatory reaction caused by the intra-prostatic reflux of urine contaminated with BCG in the bladder^3^. The hypothesis that hypersensitivity reactions to BCG antigens may cause infectious complications including BCG-induced GP has been generally acknowledged^8,9^. It can occur in normal, carcinomatous, or, more commonly, hyperplastic prostate glands. Various predisposing factors such as UTIs, surgical interventions including transurethral resection of the prostate and prostatectomy, needle biopsy, and instillation of BCG into the bladder are known to be associated with GP^3,8,10^.

The clinical and radiological progression of BCG prostatitis resembles the changes of prostate cancer and may pose challenges in patient management. Digital rectal examination (DRE) may reveal nodules on the prostate gland and ultrasound imaging may show abnormal findings, which represent underlying pathological changes^4^. Along with elevated serum PSA levels, these findings may suggest the probability of prostate cancer.

In a study done by Leibovici *et al*., 75% of their study population had elevated serum PSA levels. The increase was clinically significant (>4 ng/mL) in approximately 40% of the patients, but was a transient event and serum PSA levels returned to normal within 3 months^4^. Another recent study found that while 40% of their BCG administered patients had PSA levels greater than 4 ng/mL after BCG induction, approximately 45% of the patients showed high PSA levels at 3 months from the start of therapy^11^. Difficulties in patient management may be caused by abnormalities in MRI^12,13^ or by unusual F-18 FDG uptake in the prostate during positron emission tomography-computed tomography scans^14^. In such situations, Leibovici *et al*. suggested monitoring PSA levels and avoid prostate biopsy in patients treated with BCG^4^. Moreover, Beltrami *et al*. encouraged conservative management using follow-up serum PSA levels and delay biopsy of the prostate for a year^11^. Patients with a PI-RADS score of 3 or less on a MRI scan should be considered for a follow-up radiologic studies, rather than undergoing prostate biopsy^13^. Patients with lesions with a higher PI-RADS score may need to be assessed by a biopsy to exclude prostate carcinoma.

In this study, there was PZ involvement in 66% of the cases, while a smaller number of lesions (34%) were identified in the TZ. This finding is consistent with a study that compared radiologic and pathological findings of 6 patients who underwent BCG therapy^15^ and with study results done by LaFontaine *et al*. that documented the occurrence of granulomatous prostatitis and the presence of acid-fast bacilli^2^.

Miyashita *et al*. noted that BCG prostatitis formed wedge-shaped granulomatous areas in the PZ, where the wedge formation started from the urethra and spread out in the direction of the prostatic capsule. The granulomas formed in proximity to the duct-acinar system and were observed in the lumen, duct wall, and periductal areas adjoining larger caseous granulomas^15^. This specific distribution of BCG prostatitis implies that the urine reflux into ducts draining the PZ was a factor in the pathogenesis of this entity. Moreover, after studying post-mortem and *in vivo* specimens, Kirby *et al*. concluded that reflux of foreign particles in prostatic ducts is a possibility; frequently in the PZ^16^.

The association between prostate volume and BCG-induced prostatitis shown in this study could suggest that prostate hyperplasia makes it a larger target for infection. Currently, uncertainty remains as to whether BCG granulomas are a consequence of enlargement and further investigation into causality will require additional studies.

Patients with chronic prostate inflammation have been shown to have larger prostate volumes, along with more severe lower urinary tract symptoms and a higher probability of acute urinary retention^17^. Recent studies strongly suggested that benign prostatic enlargement is an immune inflammatory disease. The T-cell activity and associated autoimmune reaction induce epithelial and stromal cell proliferation^18^. Moreover, various interleukins and other inflammatory cell cytokines that are secreted by the stroma play a pivotal role in the promotion of the autocrine or paracrine proliferation of benign prostatic hyperplasia (BPH) cells^19^. BCG instillation may induce prostatic inflammation, which in turn may lead to tissue damage and continuous wound healing, thereby contributing to the formation of GP.

Evidence from prior studies consistently shows that increased adiposity was positively associated with prostate volume measured by ultrasound and MRI. An increase in prostate volume was a strong predictor of adverse clinical outcomes associated with BPH, including acute urinary retention and renal failure^20,21^. Moreover, multiple studies have shown that body weight^22^, BMI^22,23^, and waist circumference^22,24^ were positively associated with prostate volume. In a study by Parsons *et al*., the study cohort showed that a 1-kg/m^2^ increase in BMI corresponded to a 0.41-cc increase in prostate volume^22^. These observations suggest that adiposity was linked to prostate growth and therefore makes the prostate susceptible to inflammation and the development of BCG prostatitis.

There were several limitations to our study. As it was performed in a retrospective manner, the results might not reflect the exact incidence of BCG-induced prostatitis. The sample size of the study was limited; therefore, further studies are necessary to confirm the characteristics of patients with NMIBC with abnormal MRI findings of the prostate gland after BCG instillation. Follow-up MRI protocols were not standardized and the majority of the patients did not have a dedicated prostate MRI. In addition, there were difficulties in comparing the MRI findings with histological results of GP, since a prostate biopsy may increase the risk of unnecessary complications and was not performed in asymptomatic patients or those without clinical implications.

In bladder cancer patients with abnormal findings on follow-up imaging, the physician must consider the likelihood of a BCG reaction. When there is clinical suspicion for primary tumor or metastasis, a prostate biopsy may be needed to provide a final diagnosis. For patients with clinical symptoms suggesting infection, empirical therapy using tuberculosis medication with or without steroids should be administered. Infection complications are generally treated effectively with a tuberculosis drug regimen, and hypersensitivity reactions are highly responsive to steroids^25^. Follow up imaging of these patients after management for re-evaluation of imaging abnormalities would be beneficial for confirming the clinical diagnosis and excluding tumor or metastatic progression.

## Conclusions

The prevalence of post-BCG-induced prostatitis was higher in men with a higher BMI and larger prostate volume, and specific management plans should be developed for this group of patients who have an increased risk. However, our results must be interpreted with caution due to the low number of patients. Clinicians should be aware of the aforementioned risk factors when using intravesical BCG therapy to treat patients with NMIBC so that appropriate treatment can be administered and avoid unnecessary invasive procedures.
